# Gap between college education and clinical practice: Experience of newly graduated nurses

**DOI:** 10.1002/nop2.409

**Published:** 2019-11-05

**Authors:** Ji Eun Lee, In Ok Sim

**Affiliations:** ^1^ Department of Nursing Seoul National University Bundang Hospital Gyonggi-do Korea; ^2^ Red Cross College of Nursing Chung‐Ang University Seoul Korea

**Keywords:** clinical practice, college education, neurology ward, newly graduated nurse, qualitative research

## Abstract

**Aims:**

To examine the experience of new nurses working in the neurology ward and the differences between their training and hospital practice.

**Design:**

Qualitative.

**Methods:**

The interviews were conducted from 15 July–28 August 2017, in a quiet and comfortable meeting room at the hospital. Data were collected through interviews with 12 newly graduated nurses working at a neurology ward.

**Results:**

The results yielded four themes: “ineffective learning of nervous system theory in college,” “difference between learning theory and practical application,” “difficulty in nursing intervention for various clinical symptoms” and “need for teaching method based on actual cases.”

**Conclusion:**

The findings can be used for the development of effective nursing education programmes to reduce the gap between theory and practice.

## 1 INTRODUCTION

The curriculum of nursing education is operated for the purpose of fostering professional nurses who can provide quality nursing care in the field of practice by integrating theory and practice (Bland, Topping, & Wood, 2011; Jho, 2014). Nursing is practical learning that enables the practitioner to provide nursing care based on their knowledge, and nursing has significance as a discipline when knowledge is applied in actual practice after education (Goberson & Oermann, 2009). Differences in theory and practice are one of the nursing issues being discussed in many ways, such as a thread that has not been solved for a long time (Leducq, Walsh, Hinsliff‐Smith, & McGarry, 2012). Almost all of the nursing education leaders in the United States believe that new nurse graduates are adequately prepared to enter the workforce and practice; nearly the same number of nursing leaders in the hospital setting, however, disagree (Berkow, Virkstis, Stewart, & Conway, 2008). In addition, Del Bueno (2005) pointed out that the nursing curriculum is not focused on applying or using knowledge in clinical practice and also reported that most of the new nurses have difficulties or are not able to transfer knowledge to the patient care settings because they lack clinical experience.

The transition from a nursing college student to a nurse is a tough process, with many factors to overcome and many factors of stress (Bennett, 2017; Henderson, Ossenberg, & Tyler, 2015). Newly graduated nurses face a variety of challenges and enormous pressures that they have never experienced (Bennett, 2017) and have many transition issues affecting their performance (Duchscher, 2009). Moreover, they experience the shock of being prepared to become a professional nurse at a nursing college for several years, but not at all prepared after joining a medical institution (Martin & Wilson, 2011).

Most nursing students consider the nervous system a challenging subject; newly graduated nurses are pressured and stressed when placed in a neurology ward. Kim and Kang (2013) reported that senior nursing students have particular difficulty in neurological nursing. Nurses in the neurology ward mostly need to look after patients in critical condition and require the ability to deal with neurological emergencies, such as seizures. Moreover, changes with neurological conditions can occur rapidly and cause irreversible damage, so nurses should be able to perform neurological tests quickly and accurately to assess patients’ neurological state. Thus, neurology ward nurses who need to perform complicated and difficult tasks may experience lowered self‐esteem and fail to adapt to reality when they cannot apply their knowledge and skills to provide appropriate care, which may lead to a high rate of turnover.

Learning transfer refers to applying the knowledge, skills and attitudes learned through education and training into the workplace, as well as applying, generalizing and maintaining new skills and knowledge (Holton, Bates, Seyler, & Carvalho, 1997). Determining the success of an education programme requires examining the extent to which the knowledge has been efficiently used in the workplace to raise the quality of work performance and checking the extent to which one feels that his or her performance has improved; these factors are affected by the amount of learning transfer (Kim & Kwon, 2014).

The nursing curriculum is performance based. It is therefore important to understand how school education helps new nurses perform actual nursing. Thus, the effective application of the knowledge, skills and attitudes acquired at the nursing college to clinical practice merits research, especially with respect to the parts that graduates have difficulty adapting. There are some aspects of the quantitative study that are difficult to obtain a holistic view and stance about learning transfer. Thus, a research method that incorporates in‐depth interview is suggested for investigating the learning transfer of new nurses in neurological wards.

This study sought to investigate how nurses who have worked for <1 year in the neurology ward experience the process of adapting to the practice after completing their school education.

## 2 THE STUDY

### 2.1 Aims

This research aimed to examine the process of learning transfer from nursing college to clinical practice at the neurology ward among newly graduated nurses. The research question of this study was as follows: “What were the experiences in learning transfer of neurological nursing education to clinical practice?”

### 2.2 Design

This research was a qualitative study that used focus group interviews to shed light on the experiences of learning transfer from nursing education in college to clinical practice among newly graduated nurses in neurology wards.

### 2.3 Participants

The participants were 12 newly graduated nurses with less than a year of clinical experience in neurology wards located in university hospital.

### 2.4 Data collection

To deeply understand the new nurses’ experience from nursing education in college to clinical practice, focus group interview was used to collect data.

Focus group interview as a qualitative method is used to gather data from a group with similar characteristics. It is conducted by encouraging a discussion among participants to obtain knowledge of their deep thoughts and ideas on a specific topic (Krueger & Casey, 2014). Unlike in‐depth interviews, focus group interview can draw data that are difficult to derive from other methods by promoting social interaction between the participants (Morgan, 1996). All the subjects were new graduate nurses working in neurological wards, so they could freely share their thoughts and experiences in a focus group.

A researcher conducted the focus group interviews. The researcher, who is a nurse in the neurological ward, has worked in the neurological ward for 11 years and has a deep understanding of the neurological ward nursing. In addition, researcher has several preceptor experiences, educated new nurses in the neurological ward and trained new nurses to the core nursing skills. The researcher, however, is a general nurse who has no impact on the evaluation or employment of new nurses. The researcher explored theoretical bases for conducting the qualitative study, reviewed the related literature to build theoretical groundwork and learned attitudes and methods by attending “qualitative research methodology” during graduate courses and conference on qualitative research.

The researcher explained the purpose, method and duration of the study to the participants before the interviews were held. The participants were given the right to withdraw from the research at any time, and voluntary informed consent was acquired prior to their participation. The focus group interviews were set according to the several participants’ work schedules; they were contacted through information they had provided. The participants had three work shifts; accordingly, three groups were organized with three to four people in each.

The interviews were conducted from 15 July to 28 August 2017, in a quiet and comfortable meeting room at the hospital. They were carried out for about 100 min per group and were recorded using digital recorders. Non‐verbal expressions were used to show that the moderator was actively listening, and follow‐up questions were incorporated if necessary. After the third focus group interview, data collection was stopped, as much of the content was repetitive and no new data could be identified. No participant withdrew from the study. As soon as the interview ended, the contents were transcribed so that the experience was still fresh in the interviewer's mind and the recording was repeatedly played to confirm the accuracy of the transcription.

### 2.5 Ethical Considerations

The study was approved by the Institutional Review Board (IRB no. B‐1705‐397‐301) of “B” Hospital, located in Gyeonggi Province, for the ethical protection of the participants. The researcher, who had no influence on the evaluation of the new nurses’ performance and employment, thoroughly explained the purpose, method and duration of the study to the participants, along with issues relating to confidentiality, anonymity and protection from harm.

### 2.6 Data analysis

The data were analysed through the content analysis method developed by Krueger and Casey (2014). The recorded contents were listened to repeatedly and read the contents of the warrior in depth. The learning contest was unitized without overlapping contexts related to the course experience and records, and coding were performed after sampling process to support the subject. Various interpretations of the data have led to the simplification of re‐examination and summation to prevent confusion and to clarify the course in the learning war. The answer to the research question was derived as an epic step that expresses the results of the study so that others can understand the results of the study after conducting a spasmodic reasoning that links the descriptive description of the course with its meaning. Through this process, 149 meaningful statements were extracted and 426 meaning units and 12 categories were derived to the final four themes.

### 2.7 Rigour

To establish validity and reliability, the researcher applied Lincoln and Guba (1985) criteria for truth value, applicability, consistency and neutrality. All interviews were recorded, and verbatim transcription was done. Truth value was confirmed by first ensuring that the recording and the transcribed text match each other and then by having one of the study participants read what was analysed and agree that it coincides with her experience. In case of applicability, one new graduate nurse and an experienced nurse in neurological wards, where they were not the participants, read the results of the study and agreed that those experiences were significant discussion. In addition, consistency was maintained by an experienced nursing professor through a general assessment of the results, while neutrality was obtained by understanding the data by eliminating any biases or stereotypes.

## 3 FINDINGS

### 3.1 General characteristics

All participants in this study were female nurses with an average age of 24.2 years. All participants were placed in the neurology ward as new nurses and had worked for <8 months.

### 3.2 Experience in the neurology ward

In analysing the participants’ experiences, 426 meaningful concepts were derived, which were classified into 12 categories. Ultimately, four themes were derived: “ineffective learning of nervous system theory in college education,” “difference between learning theory and practical application,” “difficulty in integrating nursing interventions for various clinical symptoms” and “need for teaching methods based on neurological cases” (Table 1).

**Table 1:** Themes and Categories Derived from Newly Graduated Nurses’ Experiences of Learning Transfer in the Neurology Ward

Theme	Category
Ineffective learning of theories on the nervous system in college education	Lack of quality and quantity of learning on the nervous system
	Insufficient clinical practicum to shift from theory to practice in the neurology ward
	Neurological theory difficult to learn in depth owing to decreased interest
Difference between learning theory and practical application	Experiencing a theory–practice gap when performing neurological assessment
	Difficult to apply what is learned in college
	Feeling the limits of learning about neurology
Difficulty in applying nursing interventions for various clinical symptoms	Difficulty in conducting various complex methods of assessment
	Difficulty in recognizing neurological deterioration
	Overall evaluation ability and lack of performance
Need for training methods based on neurological cases	Hoping for education that reflects complex clinical scenarios
	Wanting to get an education delivered through various methods of teaching
	Hoping for education that fosters integrated skills

#### 3.2.1 Ineffective learning of nervous system theory in college education

The participants in this study reported that theoretical learning in the undergraduate curriculum of the neurology nursing department was ineffective. Regarding the neurological theory part, the participants said that the related illnesses were too complicated and difficult to understand and that it was not only difficult to learn independently but also allotted little time and few lectures. In this study, only six out of the 12 participants (50%) reported having experienced practicum in a neurology ward. However, during the course of learning, the participants found it difficult to nurse patients with complicated instruments, such as operating monitoring devices and EKG. Further, patients with serious conditions, especially unconscious patients, were difficult to communicate with. Nevertheless, the participants who had experience in neurology ward training reported that such training helped them overcome their fear when working as a new nurse in the neurology ward.

For the nurses who work in the neurology ward after graduation, there is not enough learning to be able to make the transition from education to practice. This lack of learning indicated that working as a nurse in the hospital carried the pressure to start relearning.

In addition, the participants reported a lack of opportunity in their undergraduate programme to experience clinical practice that can help them understand and learn the nursing practice related to the neurological system and neurological diseases. Particularly, the participants who had experience in nursing ward training as students shared that the symptoms of the nervous system in relation to diseases and the patients’ symptoms were complicated and difficult to understand and there was a pervasive negative thought about nursing them. For this reason, when starting as a new nurse in the neurology ward, most of them did not have the relevant theoretical basis and could not apply knowledge to nursing for clinical cases. Participants explained that working as a new nurse in the neurology ward was so stressful that they constantly thought about quitting:

When I was a student, I did not have much chance to study deeply about neurological theory and I did not even have a practical experience. When I started working in a neurology ward, I had no idea what to do for the patients. (Group 2‐3)

It was so shocking when I had a practicum at NCU. At the time, I had limited knowledge and there was no one explaining in detail why this patient had these devices… [everyone was] all too busy. The patient that I can remember right now is one who was confused, spitting and saying nonsensical stuff. (Group 2‐2)

Some participants had negative thoughts on nursing after seeing craniectomy patients with opened skulls, patients with many monitoring devices attached to them and unconscious patients:

During the week I spent on practicum, [it seemed] patients were attached to all of the monitors that they could possibly need […] [such as] EKG monitors […] and devices that recorded brain waves. [It was] too scary. [I] felt like I could not even touch them. (Group 4‐1)

#### 3.2.2 Difference between learning theory and practical application

The participants learned about the theory and practice of neurology in school, but had difficulty applying the method in clinical practice. Regarding the approach to learning at school, they reported that there were many differences between school and application in the neurology owing to the knowledge related to the nervous system that they acquired. They also indicated the difficulty in performing the learning transition owing to the complexity of consciousness levels and emergency situations arising from changes in patients’ conditions.

For example, as new nurses, the participants stated that even basic clinical practices, such as the Glasgow Coma Scale and motor power assessment, were difficult to perform. In observing external ventricular and lumbar drainage for the first time, they recognized their insufficient theoretical basis for administering management and preventive measures. It was difficult to perform owing to their fear of making mistakes. In this way, the theories taught as prior learning at school had limitations in terms of applicability to clinical practice; the knowledge and skills gained at school were no longer sufficient for clinical practice:

Learned about seizures in school, but i did not know how and what to do first when my patient had a seizure. (Group 2‐3)

When I was learning at school, I was only taught fundamental nursing skills, without special cases; [the lessons] focused on protocols in a repetitive manner. However, if I had been given situations that reflected the actual characteristics of patients with neurological disorders and learned more about how to perform those skills, then I think I could have used them better. (Group 4‐1)

I learned in school that the Glasgow Coma Scale is a way of assessing the level of consciousness, but I did not know how to administer it and the concept was new as it was not related to theory and practice. (Group 3‐3)

It was my first time seeing external ventricular drainage here. I did not even know how to begin [my task] because [I was afraid] I might make a mistake and things may go wrong. It involves putting a very important piece of equipment into the patient’s body. (Group 4‐2)

I remember I learned about intracranial hypertension. I have this kind of memory, but I can’t remember it in the actual clinical setting and I didn’t even try to recall, because I felt it was different in clinical practice. (Group 2‐1)

#### 3.2.3 Difficulty in integrating nursing interventions for various clinical symptoms

Participants reported difficulty in performing integrated nursing interventions for acute chronic symptoms of neurology patients based on only their education in nursing college. Nursing students study only the diseases classified by body organ; thus, they could be unaware of the interconnected symptoms. Basic nursing is important for these problems, and it is critical to acquire comprehensive assessment ability and learn the performance method for neurology patients with various characteristic symptoms. The ability to intervene using integrated nursing is explained by the ability to understand and diagnose the relation among components of a whole system when assessing the patient's problems based on rationale. The problem is that nursing education requires not only piecemeal knowledge but also the ability to integrate such knowledge into a higher level.

The participants reported that during their work in the neurology ward, they had many difficulties in evaluating neurological conditions, an essential technique for identifying patients’ problems. Therefore, it is necessary to strengthen the ability to perform nursing, which requires assessing the neurology patients and identifying problems. In school education, nursing educators should use scenarios to explain actual situations rather than simply using textbooks to convey knowledge to students. Instructors should provide information on subjects using video, nursing plans and mediation processes to solve problems:

When working in a neurosurgery ward, I found that the condition of the patient did not cause any particular problems but actually worsened suddenly. Therefore, when working as a nurse, sharp insight is needed because the condition of consciousness is assessed and nursing without intensive attention can cause a very serious condition to be overlooked. (Group 4‐3)

I studied hard about blood tests and normal ranges at school. At that time, I thought, “Why do I have to memorize these?” In recent years, however, I have tested ammonia in patients with seizures and the results were very high; I remembered what I learned at school. I knew this test result could happen because of the decrease in consciousness and medicine (when the results were high), but it was difficult to apply the whole nursing process regarding what to do next. (Group 3‐1)

I was not able to communicate with the patient and suddenly, I did not know how to assess and find a solution when their condition worsened. (Group 3‐4)

It is difficult to assess the patient’s overall illness and it is difficult to assess the patient comprehensively, considering the fact that older adult patients suffer from hypertension, diabetes and hyperlipidemia. (Group 1‐1)

When I am nursing a patient with a neurological problem, I do not always feel confident when I need to decide on an integrated nursing assessment and mediation method because I know only partial theory about brain diseases, their relation with the body, [and] the symptoms of the patient. (Group 2‐2)

#### 3.2.4 Demand for teaching methods based on actual neurological cases

The participants were asked about changes to the teaching methods to reduce the gap between nursing education and clinical practice in the process of learning transfer. The current schooling emphasizes basic knowledge of diseases, and so, there is no opportunity to develop a practical and integrated capacity to nurse patients and their families. Despite the importance of the current learning method, theory and clinical practice experience, the necessity to have an education method that recognizes the actual situation and mitigates difficulties in mediation should be highlighted.

Participants suggested that learning methods using scenarios in actual situations, teaching methods through simulations and videos and methods using app‐based programs could help reduce the gap between theory and practice. In addition, they stated needing a learning method that allows students to acquire an integrated approach to nursing intervention by linking the education acquired in the clinical situation with actual real situation rather than focusing on only detailed knowledge of each concept.

More effective learning methods are important for improving the nursing ability of new nurses working in areas such as neurology wards. The participants explained the importance of a systematic, case‐based education method that enables the student to experience situations indirectly, such as various diseases and symptoms, rapidly changing consciousness problems and communication problems.

Therefore, the structure and method of school education need to be improved. Meanwhile, students should actively and flexibly cope with school education to deal with complicated and various situations that can occur in the clinical environment:

Seizures are a common symptom for patients in the neurology ward. I first saw a patient who suddenly had a seizure while working in a ward. I could not cope … I think I would not have been so embarrassed if I had been given simulation training on such situations. (Group 4‐2)

Problem‐based learning improves critical thinking skills and is more clinically beneficial than lecture‐type teaching methods for theoretical instruction. (Group 1‐2)

There was a professor who taught me how to think. For example, a person suddenly collapses, their eyes floating and mouth full of saliva. Why do you think this person is like this? Which nursing interventions [are needed] to manage this? At first, I thought it was a seizure but it was not a seizure but syncope. With such teaching methods, I gained a broader perspective in thinking in the clinical setting. (Group 4‐2)

During simulation training, I was assigned to different roles and I had the opportunity to practice like a real nurse and a patient. Through this simulation, we were able to learn both the patient’s and nurse’s roles. (Group 2‐2)

I saw a video of the actual situation of the patient’s symptoms and problems. [This method] also lets students have fun with each other, discussing issues on the patient’s problems and nursing interventions and thinking differently about emergency situations, such as how to medicate, what to look for when talking to doctors and nurses and how to tell others about one’s experience. (Group 4‐3)

## 4 DISCUSSION

The purpose of this study is to present the experience of new nurses working in the neurology ward with respect to the process of adapting their school learning to clinical practice. The themes analysed in this study were as follows: “ineffective learning of nervous system theory in college education,” “difference between learning theory and practical application,” “difficulty in applying integrated nursing interventions for various clinical symptoms” and “need for teaching methods based on neurological cases.”

First, one participant reported a lack of sufficient experience in the theoretical instruction on the nervous system in college education (i.e. matching the first theme of “ineffective learning of nervous system theory in college education”). Although it is important to learn about specific symptoms, such as characteristics, nerve emergencies and changes in consciousness, clinical practice needs to be given appropriate attention.

Bennett (2017) highlighted the importance of providing opportunities for a balanced and unbiased learning in all areas of study. Further, it is important for students to develop their own potential in performing professional tasks. In particular, nursing science has been reported to be limited in terms of allowing students to learn the nursing process for specific illnesses and symptoms in the curriculum within a given period (Maben, Latter, & Clark, 2006). McKenna and Wellard (2004) stated that nursing practice provides students with learning to extend theory to clinical practice. Clinical practice is an effective method for applying theory and practice; a core process of nursing education is the application of theoretical knowledge to practice.

Thus, effective theoretical instruction and clinical practice can strengthen the learning transfer and develop competencies to transform learned knowledge into a stable state. As the curriculum of nursing college is not intended for vocational school education, it is inevitable that there is a difference between the knowledge learned at school and the actual work performance. Organizational and individual efforts are needed to respond flexibly amid differences between theoretical learning and actual clinical practice.

Second, the participants noted the difficulty in applying the lessons learned in school to clinical practice. Many studies have shown that the core basic nursing skills learned through repetitive practice have a higher level of performance in clinical practice compared with pre‐learning levels (Chung, Kim, & Shin, 2016). The participants who were educated under instruction that focused on the theoretical aspect, compared with clinical studies, reported facing difficulties in applying theories to the clinical situation, especially in the neurology field. These results correspond to those of Kim and Kwon (2014) who emphasized that learning core nursing skills as in actual clinical situations would enhance confidence in the performance of new nurses and improve adaptability in clinical settings.

Third, participants reported difficulty in performing integrated nursing interventions for acute chronic symptoms of neurological patients based on their education in nursing college. For this, there is a need to develop a stepwise education programme. Simulation training through scenarios that reflect real‐life cases in clinical practice, undertaken after learning fundamental nursing skills, would not only allow students to acquire skills through repeated practice but also improve integrated and critical thinking.

Finally, the participants expressed the need for teaching methods based on neurological cases. In this study, the participants realized that nursing education based on appropriate teaching methods, such as problem‐based learning and educational simulation, which are based on cognitivism and constructivism, is effective in clinical practice. The results of this study indicated that the participants wanted changes in nursing education, particularly about the appropriateness of instructional design of teaching methods. To develop nursing competencies, the participants wanted to receive a comprehensive education delivered through effective teaching methods that reflect clinical settings. The theory–practice gap can be reduced by promoting smoother transitions in the learning transfer. The same suggestion was presented in Huston et al. (2017), which proposed innovative changes in teaching methods and the incorporation of simulation training as a strategy to reduce the differences between theory and practice. Thus, efforts should be made to apply appropriate and diverse teaching methods in designing nursing education by having it centred on the learner rather than on the instructor, to ensure effective learning transfer.

The participants also wanted to learn about neurological assessment using a web‐based program, given that such assessment is difficult to experience directly. Thus, appropriate teaching methods are expected to reduce the theory–practice gap by enabling the smooth transition of learning transfer.

### 4.1 Limitations

This study was limited to the objectification of the results because it is a study conducted only in one university hospital neurological ward. So it is difficult to generalize the findings to the target population. Therefore, it is recommended for the future studies to expand its scope to new nurses who work in various medical institutions.

## 5 CONCLUSION

This study involved an in‐depth exploration of the experiences of the process of adapting college learning and clinical practice in neurological education among newly graduated nurses with less than a year of clinical experience in a neurology ward.

The participants said that educational simulation in neurology was not efficient and they had difficulty in applying what they had learned at school to clinical practice. From these experiences, the participants hoped to develop nursing competencies and learn through effective teaching methods.

The results of this study highlight the importance of nursing education. Moreover, it is even more important for new nurses to work in special wards, such as neurology wards.

Based on the results of the study, it is suggested that a plan should be developed to prepare newly graduated nurses in neurology wards by improving learning transfer. It is important to provide them with opportunities to experience and learn through various types of simulation in the course of learning transfer with reference to their theoretical knowledge. Moreover, appropriate teaching methods should be incorporated with a student‐centred approach. These results could serve as a basis for the development of effective nursing education programmes that can reduce the gap between theory and practice for new nurses.

## CONFLICT OF INTEREST

The authors declare that they have no conflict of interest.

## ETHICAL APPROVAL

The study was conducted after receiving approval from the Seoul National University Bun Dang Hospital institutional review board (1041078‐201312‐HR‐00109‐03).

## INFORMED CONSENT

Informed consent was obtained from all individual participants included in the study.
